# Is parenting style a predictor of suicide attempts in a representative sample of adolescents?

**DOI:** 10.1186/1471-2431-14-113

**Published:** 2014-04-26

**Authors:** Carolin Donath, Elmar Graessel, Dirk Baier, Stefan Bleich, Thomas Hillemacher

**Affiliations:** 1 Center for Health Services Research in Medicine, Department of Psychiatry and Psychotherapy, Friedrich-Alexander-Universität Erlangen-Nürnberg, Schwabachanlage 6, Erlangen, 91054, Germany; 2 Criminological Research Institute of Lower Saxony, Lützerodestr 9, Hannover, 30161, Germany; 3 Center for Addiction Research, Clinic for Psychiatry, Social Psychiatry and Psychotherapy, Hannover Medical School, Carl-Neuberg-Str. 1, Hannover, 30625, Germany

## Abstract

**Background:**

Suicidal ideation and suicide attempts are serious but not rare conditions in adolescents. However, there are several research and practical suicide-prevention initiatives that discuss the possibility of preventing serious self-harm. Profound knowledge about risk and protective factors is therefore necessary. The aim of this study is a) to clarify the role of parenting behavior and parenting styles in adolescents’ suicide attempts and b) to identify other statistically significant and clinically relevant risk and protective factors for suicide attempts in a representative sample of German adolescents.

**Methods:**

In the years 2007/2008, a representative written survey of N = 44,610 students in the 9^th^ grade of different school types in Germany was conducted. In this survey, the lifetime prevalence of suicide attempts was investigated as well as potential predictors including parenting behavior. A three-step statistical analysis was carried out: I) As basic model, the association between parenting and suicide attempts was explored via binary logistic regression controlled for age and sex. II) The predictive values of 13 additional potential risk/protective factors were analyzed with single binary logistic regression analyses for each predictor alone. Non-significant predictors were excluded in Step III. III) In a multivariate binary logistic regression analysis, all significant predictor variables from Step II and the parenting styles were included after testing for multicollinearity.

**Results:**

Three parental variables showed a relevant association with suicide attempts in adolescents – (all protective): mother’s warmth and father’s warmth in childhood and mother’s control in adolescence (Step I). In the full model (Step III), Authoritative parenting (protective: OR: .79) and Rejecting-Neglecting parenting (risk: OR: 1.63) were identified as significant predictors (p < .001) for suicidal attempts. Seven further variables were interpreted to be statistically significant and clinically relevant: ADHD, female sex, smoking, Binge Drinking, absenteeism/truancy, migration background, and parental separation events.

**Conclusions:**

Parenting style does matter. While children of Authoritative parents profit, children of Rejecting-Neglecting parents are put at risk – as we were able to show for suicide attempts in adolescence. Some of the identified risk factors contribute new knowledge and potential areas of intervention for special groups such as migrants or children diagnosed with ADHD.

## Background

The WHO predicts that suicide will contribute more than 2% to the global burden of disease in the year 2020 [1]*.* Thus, the prevention of suicide is considered to be a major health goal by global health politicians. The European Union supports this global prevention strategy by supporting research to enhance suicide prevention interventions (for example, the SEYLE trial: [2,3] or the OSPI Europe project: http://www.ospi-europe.com[4]). This is important when considering that suicide is currently one of the leading causes of death in Europe among young and middle-aged people [5].

In Germany, prevention projects have been implemented for adults, such as the Nuremberg Alliance against Depression [6-8] or the Freiburg Alliance against Depression (http://www.freiburger-buendnis-gegen-depression.de; [9]). There is also a National Suicide Prevention Program (“NaSPro”) in collaboration with the German Ministry for Health, the European Network on Suicide Prevention, and the WHO [10].

In all of the named prevention initiatives, it is stated that prevention is possible. Therefore, the risk and protective factors for suicide need to be known. This work focuses on a special group (i.e., adolescents) in one country (i.e., Germany). Germany presents a special case because, according to the OECD, the social status of parents has a tremendous impact on the success and development of adolescents [11,12], and the percentage of children living in poverty (16%) is among the highest in the Western industrialized countries [13]. The goal is to identify significant and clinically relevant risk and protective factors for suicide attempts in 15-year-olds. The lifetime prevalence of suicide attempts in this group is stated to be appr. 9% in Germany (Donath C, Gräßel E, Baier D, Hillemacher T: **Association between heavy episodic drinking and suicidal thoughts and attempts in a representative sample of German adolescents, submitted)**, about 10.5% on average across 17 European countries [14,15], and about 4.1% in the U.S. [16].

The focus of this work lies in identifying the role that parenting styles experienced in childhood play in adolescent suicidal behavior. Next to the association of parenting styles with suicide attempts, other potential risk and protective factors are to be identified.

### What is known?

We know that childhood experiences with parenting styles are associated with several risk behaviors and personality aspects, especially when rather “adverse” parenting styles such as the Authoritarian or Rejecting-Neglecting styles are evident [17]. For example, higher substance use, lower self-esteem, and lower social competence in adolescents are associated with Authoritarian parenting [18] in comparison to Authoritative parenting. Furthermore, we know that adolescents with Authoritative parents have significantly higher self-esteem, higher self-control, and stronger resistance to peer influence, thus reporting lower substance use and violence-related behaviors than peers whose parents are defined as Rejecting-Neglecting [18].

Concerning suicidal ideation or suicide attempts and parenting style, the literature is sparse: We know from adolescents in Hong-Kong that suicidal ideation is associated with perceived Authoritarian parenting expressed in low parental warmth and high maternal control [19]. This is supported by another study from Australia [20], where adolescents with parents high in control and low in affection (i.e., Authoritarian parenting) have double the risk of suicidal ideation and three times the risk of deliberate self-harm. It is also known that parental hostility is associated with suicidal behaviors [21] in boys in particular, where experiences with parental violence have been shown to predict suicide attempts. A study in Chile found rather weak associations between parenting styles and suicidal ideation [22]. However, there are no current studies in Germany or even Europe with data that can address this research question.

Beyond parenting styles and parenting behavior, there are already some well-known risk factors for suicidal ideation and attempts in adolescents; for example, age [23] and sex (e.g. [24]). We also know that the experience of violence, especially psychological abuse, is often a direct antecedent of suicide attempts [25]. However, this work attempts to go beyond the “well-known” factors – with the goal of creating knowledge about possible risk or, even better, protective factors for adolescent suicidal behavior.

#### Aims

I) To analyze the predictive value of parenting variables and parenting styles for suicide attempts

II) To explore other significant predictors of suicide attempts in adolescence

III) To define *relevant* protective and risk factors for suicide attempts in adolescence

## Methods

### Design

The study employed a representative survey of 9^th^ graders in Germany conducted in 2007/2008. In the year 2006, there were 910,000 9^th^ graders in Germany. The goal was to survey 50,000 adolescents from different regions. With knowledge about the number of 9^th^ graders in each class of region size (from the official education statistics) and the goal of questioning 50,000 adolescents, it was possible to calculate how many adolescents per class of region size had to be included. Note that classes were drawn by chance, but students were not. The goal was to match the distribution of the 9^th^ graders in the classes of region size (in the population) to the same percentage in the sample. It was assumed that every 2^nd^ student (in large cities, every 6^th^ student) in a drawn region would be questioned. Thus, we were able to calculate how many regions had to be drawn out of every class of region size. These steps resulted in 61 regions. Regions were then drawn by chance in order to secure a representative sample. At the Criminological Research Institute of Lower Saxony, we stratified by school type to draw the sample. Then all directors of the schools that were drawn were informed in writing about the survey and asked for the participation of their 9^th^-grade school classes. If the directors agreed to allow their students to participate in the survey, we sent informational material to the schools including consent forms for parents. The study was announced by a letter sent to the parents and to the students from the KfN. The study was not announced as a study on suicidality, since it was in reality a study with broad interest. The official announcement was “concerning different problems in the youth”. The teachers in the classroom who delivered the questionnaires referred to the information letter. There was no incentive to take part other than that two school lessons were cancelled for the time when the questionnaire was filled out.

On an appointed day, the written survey was administered to all 9^th^-grade students except for the students whose parents refused participation, who themselves refused to participate, or who were otherwise busy or absent when the survey was administered. The survey at the school was carried out by trained external study assistants – not by the employees of the schools – in order to preserve reliability and validity.

The research project was granted by the Federal Ministry of the Interior in Germany. The survey was audited by each Ministry of Education of every German state (Bundesland) and of every state responsible for data protection. The ethical commission of each participating German state’s ministry of education approved the survey. As a consequence of their vote, the survey was strictly anonymized – no names, no addresses, and no school addresses were obtained. Written consent was obtained from the parents of the adolescents. If the consent of the parent(s) was not available, the student could not participate in the survey. Furthermore, students were themselves free to decide whether they wanted to take part in the survey. If they were not willing to do so, they worked on alternative material given to them by their teachers. Two manuscripts based on this data set have already been published, and one is under consideration. These manuscripts concern epidemiological data on Binge Drinking [26,27] and the prevalence data of suicidal thoughts and suicide attempts (Donath C, Gräßel E, Baier D, Hillemacher T: **Association between heavy episodic drinking and suicidal thoughts and attempts in a representative sample of German adolescents, submitted)**.

### Instruments

The dependent variable, the lifetime prevalence of suicide attempts, was assessed with a single item developed by the Criminological Research Institute of Lower Saxony asking “Have you ever seriously tried to commit suicide?” A dichotomous “yes” or “no” answer was the result. In a sensitivity analysis, “suicidal thoughts” was used as the dependent variable. This was assessed with the single item “Have you ever had suicidal thoughts?” The item was constructed by the Criminological Research Institute of Lower Saxony. For the analyses, the item was dichotomized as “yes/no” with “no, never” recoded to “no” and “yes, rarely”, “yes, sometimes”, and “yes, often” recoded to “yes”. The dichotomous dependent variables were coded 0 (no) and 1 (yes).

#### Parental behavior/parenting styles

Parenting behavior was assessed in detail with eight variables (parental warmth and parental control in childhood and adolescence, assessed for fathers and mothers). With that information, summative variables were constructed according to Baumrind’s [17] four well-known parenting styles: Authoritative, Permissive, Authoritarian, and Rejecting-Neglecting.

Parental behavior:

▪ Parental warmth in childhood

A scale based on the concept of parenting style by Baumrind [17] (translated by Wilmers et al. [28]) was used. It consists of six items exploring parental warmth in childhood for the mother and father separately. The students were asked to think of the time before they were 12 years old when they answered the items. Cronbach’s alphas for the scale were .86 (mother’s warmth) and .90 (father’s warmth).

▪ Parental control/supervision in childhood

A scale based on the concept of parenting style by Baumrind [17] (translated by Wilmers et al. [28]) was used. It consists of three items exploring parental control and supervision by the mother and father separately. The students were asked to think of the time before they were 12 years old when answering the items. Cronbach’s alphas were .66 (mother’s control) and .77 (father’s control).

▪ Parental warmth in adolescence

The same six parental warmth items were used, but the adolescents were asked to answer the questions for the time-frame of the last 12 months. Cronbach’s alphas for the scale were .89 (mother’s warmth) and .90 (father’s warmth).

▪ Parental control/supervision in adolescence

The same three parental control items were used, but the adolescents were asked to answer the questions for the time-frame of the last 12 months. Cronbach’s alphas for the scale were .76 (mother’s control) and .80 (father’s control).

Parenting styles: According to the suggestion by Baumrind [17], only the four variables of parental warmth and parental control in childhood (mother and father) were used for computing parenting styles. Parental warmth (control) was computed as the mean of the variables mother’s and father’s warmth (control). Families were classified as “high” in control (warmth) when their scores were half a standard deviation or more above the overall mean and “low” when the scores were half a standard deviation or more below the overall mean. This algorithm was suggested by the original author of parenting styles, Diana Baumrind [17]. Persons classified “high” in warmth *and* control received the label “Authoritative”, persons classified “high” in warmth but “low” in control were labelled “Permissive”, “low” in warmth but “high” in control led to the parenting style “Authoritarian”, and a classification of “low” in both warmth *and* control was labelled “Rejecting-Neglecting”. For each adolescent (case), there were thus four variables with a dichotomous format: Authoritative parenting yes/no; Authoritarian parenting yes/no; Rejecting-Neglecting parenting yes/no, and Permissive parenting yes/no.

The following paragraphs describe the variables that were chosen as possible predictors of suicidality.

1. Age: Participants were asked “How old are you?”

2. Sex: The adolescents were asked “What is your sex?”

3. Migration background: Migration background was defined as having at least one parent who was born outside of Germany, having been born outside of Germany oneself, having non-German citizenship, or having at least one parent with non-German citizenship. The birth place and citizenship of the adolescent and his/her parents were included in the questionnaire. A summarizing variable with four categories was computed: I) German (no migration background), II) Eastern European (all countries of the former Soviet Block, former Yugoslavia, and other Eastern European countries), III) Islamic imprinted countries (all countries whose culture is essentially influenced by Islamic theology), IV) other countries (Western and Southern Europe, Christian-theology-influenced Africa, North America). The classification that “Islamic imprinted countries” are analyzed as a separate group came into existence because of our already undertaken analyses concerning other risky behaviors for example in the substance consumption field. We observed that adolescents with roots in those countries behaved obviously different; while there was no big difference between adolescents with migration background from different countries with a rather “western” culture.

4. Welfare status: The students were asked whether their parents or they themselves lived on social welfare (receiving unemployment or “Hartz IV” welfare aid according to German social legislation).

5. Parental separation events: The students were asked whether their parents were separated or divorced or whether their mother or father had died. If one of the items was answered yes, the student received a “positive” parental separation score.

6. Binge Drinking: The item assessing heavy episodic drinking (Binge Drinking) was derived from the representative survey of adolescents of the German Federal Center for Health Education [29]. Binge drinking is defined as the consumption of five or more standard drinks at one drinking occasion. For the analyses, the variable was dichotomized as Binge Drinking “yes” (5 or more drinks on at least one day of the last 30 days) or “no”.

7. Smoking (12-month prevalence): The students were asked “How often in the last 12 months did you smoke cigarettes?” The item was dichotomized; constructed by Wetzels et al. [30].

8. Non-profit volunteer activities: The students were asked for six different non-profit volunteer activities (e.g., working as a trainer for children) concerning their current involvement. An involvement score was built across the six areas.

9. School grades: A mean school grade was computed for the three self-stated school grades in Math, German, and History. Because of the ordinal data structure, the median was used.

10. Social integration in school: The extent to which a student is integrated and accepted at school was assessed with two items asking for a self-rating of one’s popularity with other students and the self-rated estimation of having a lot of friends at school. A sum score of the two items was used.

11. Absenteeism/Truancy: Students were asked to indicate whether the item “I have so far never been truant a whole day” was true for them. All students who did not check the item received a “positive” truancy score. The item was constructed by Wilmers et al. [28].

12. Attention deficit hyperactivity disorder (ADHD): The student had to answer whether a psychologist or a doctor had ever diagnosed an attention deficit disorder.

13. Self-esteem: The construct was assessed with a scale developed by Ravens-Sieberer et al. [31] and is part of the KINDL questionnaire, which assesses health-related quality of life in children and adolescents with a total of six dimensions. The dimension self-esteem consists of four items with a Cronbach’s alpha of .61. Higher scores indicate higher self-esteem.

14. Mental well-being/mood: The construct was assessed with a scale developed by Ravens-Sieberer et al. [31] and is also part of the KINDL questionnaire. The dimension mental well-being/mood consists of four items with a Cronbach’s alpha of .56. For this scale, higher scores indicate lower well-being.

15. School anxiety: The construct was assessed with a scale developed by Wilmers et al. [28] consisting of five items with an internal consistency measured with Cronbach’s alpha of .79.

### Sample

**Figure 1:**
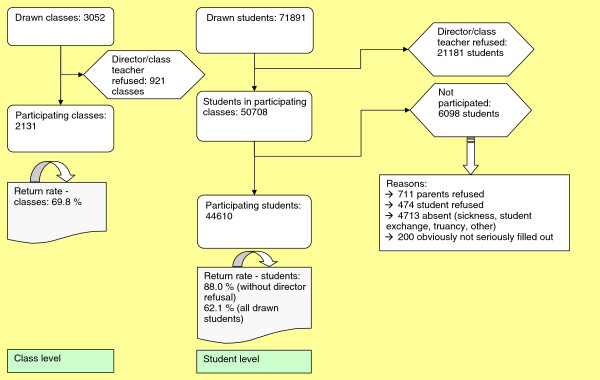
Sample constitution.

A total of 3,052 classes (9^th^ grade) with 71,891 students were drawn. For 921 classes (21,181 students), the directors/main class teachers refused to participate. 2,131 classes participated with a total of 44,610 students. Actually, the 2,131 classes included 50,708 students, but 6,098 of them did not participate (example reasons: parents’ refusal or absenteeism). Figure 1 comprises a detailed flow-chart of the evolution of the sample.

The return rates (students with director acceptance) differed between the school types and across the classification groups of region size. In spite of the varying return rates in the different classification groups of region size, the final sample represented the proportions of the population very well (e.g., students living in cities with more than 100,000 inhabitants in Western Germany: 12.04% in the sample and 11.68% in the population). The proportion of students in the 9^th^ grade in every classification group of region size in Western and Eastern Germany was compared to their proportion in the sample. With those two percentages for each category, the reliability can be rated. The proportions never differed more than 0.36% between population and sample in the different classes of region size except for Berlin where the difference was 0.62%.

To address the varying return rates, weighting factors were calculated so that the proportion of school types in the sample corresponded to that in the population, and in the same manner, the proportion of regions with different sizes in the sample corresponded to the population proportion. The two weighting factors were multiplicatively connected when the data from the total sample were analyzed. Thereby, the imbalances regarding the school types were eliminated as were the much smaller imbalances regarding the classes of region size.

The sample can be characterized as follows: 51.3% of the sample was male, the mean age was 15.3 (SD 0.7) years. The percentage of adolescents with a migration background was 27.4%, whereby students with a Turkish emigration background constituted the largest group (6.0%; more than 2,600 students) followed by emigrants from the former Soviet Union states (5.8%; more than 2,500 students). A total of 12.2% lived in large cities with more than 500,000 inhabitants including Berlin, whereas the majority lived in rural districts (68.8%). The percentage of participants with a migration background varied between 39.9% in large cities with more than 500,000 inhabitants and 23.9% in rural districts.

### Statistical analysis

We chose a stepwise analytical approach to answer the research questions (Aims I to III). The first two steps are preparing the final analytical step which is the relevant one for the interpretation of the results.

First, two basic models including either the eight parenting variables or the four parenting styles, adjusted only for age and sex, were analyzed according to their predictive value for suicide attempts. Binary logistic regressions were chosen with 0 (no suicide attempt) and 1 (positive life-time prevalence suicide attempt) as coding for the dependent variable.

Second, the influence of other potentially significant predictors (in addition to parenting style) was tested in a bivariate model with basic control variables This means that for each potential predictor (e.g., Binge Drinking, Social Status, etc.), a separate binary logistic regression analysis was computed with the control variables age and sex, the four parenting styles, and suicide attempts as the dependent variable. (The same process was carried out for the eight parenting variables but is not included in the manuscript for reasons of clarity).

In the third – final - step, all significant predictors in the bivariate models were analyzed together with the parenting style variables and the control variables age and sex in a multiple binary logistic regression with suicide attempts as the dependent variable. All variables that were checked for their bivariate relations turned out to be statistically significant; thus, the number of variables was not reduced in Step III.

Before carrying out the multivariate analysis of predictors of suicide attempts (Step III), all potential independent variables (i.e., significant variables from the bivariate analysis) were analyzed for multicollinearity. The goal was a model that was as lean as possible but still well operationalized. We determined that variables with a medium (r > .5) or even high (r > .7) correlation with other variables needed to be reduced because of redundancy in informational content. Correlation coefficients were computed according to the measurement level of the variables. As a result of the multicollinearity analysis, no variable was omitted from the multivariate analysis. The highest association was found for the variables “Binge Drinking” and “Smoking” (r = .390). As an aside, the eight parenting variables chosen as predictors in Step I were correlated with each other up to .689. This was a second reason – next to clarity and the sparse use of variables – to use the four parenting style variables as predictors in Steps II and III instead.

This means that the remaining 15 variables plus the four parenting style variables were included as predictors in a multiple binary logistic regression analysis with suicide attempts as the dependent variable. As a sensitivity analysis, this binary logistic regression described above was also carried out with the dichotomous “suicidal thoughts” variable as the dependent variable.

We applied the following procedure to cover the three analytical steps: The independent variables were included in the regression equation by the enter method. As a measure of variance explained by the model, we used Nagelkerke’s R^2^. Statistical analyses were performed with PASW 18.0. Because of the sample size, the level of significance was set to p < .001 [32]; however, we should note that statistical significance is not equivalent to clinical relevance, especially in large samples [33-35]. Therefore, the Odds Ratios and their confidence intervals were also used in the interpretation of the results. We decided to interpret a predictor as clinically relevant in our study if the Odds Ratio was higher than or equal to 1.2 or smaller than or equal to 0.8 in combination with a p-value below .001. Predictors that changed the risk in the range of at least 1.1 to 1.19 respectively in the range of 0.81 to 0.9 at a significance level of p < .001 were further considered to be on the threshold of clinical relevance. We have used and published this classification method before for predictors of Binge Drinking [27].

Missing values were evident in less than 5% of the cases across the chosen variables, (with the exception of fathers’ parental behavior). However, we chose to impute the missing values in order to include the full sample in the regression analysis and to avoid changing sample sizes across or within the three different analytical steps. The only variable that was not imputed was the variable sex (missing values 1.1%). Thus, the available sample was reduced from 44,610 to 44,134 for all analyses.

## Results

### Descriptives

The rate of suicide attempts (lifetime prevalence) was 9.0%. The prevalence of suicidal thoughts was 39.4% (5.2% often, 10.4% sometimes, and 23.8% rarely).

### Step I: basic models

In an examination of the eight variables describing parental behavior and controlled only for age and sex, a binary logistic regression (Chi^2^ (10) = 2397.307; p < .001) showed that three parental variables showed a relevant association with suicide attempts in adolescence: Motherly as well as Fatherly warmth in childhood and Motherly control in adolescence. All three of them had a protective effect when interpreting the ORs, which ranged from .81 to .87. According to Nagelkerke’s R^2^, the model explained 11.8% of the variance. Next to the three parental behavior variables, the two control variables age and sex were also significantly associated with suicide attempts, indicating a risk for females that was three times higher than for males and a positive correlation between age and number of suicide attempts (Table 1).

**Table 1:** Basic model: predictive values of eight parental behavior variables on suicide attempts (N = 44,134)

	**Regression coefficient β**	**Standard-error**	**Wald**	**df**	**p**	**OR**	**95% Confidence interval for OR**	
							**Lower value**	**Upper value**
Motherly warmth childhood	-.211	.035	37.199	1	**<.001**	.810	.757	.867
Motherly control childhood	-.026	.033	.616	1	.432	.975	.914	1.039
Fatherly warmth childhood	-.186	.033	31.915	1	**<.001**	.830	.778	.886
Fatherly control childhood	-.040	.029	1.876	1	.171	.961	.908	1.017
Motherly warmth adolescence	-.081	.034	5.821	1	.016	.922	.863	.985
Motherly control adolescence	-.141	.030	22.220	1	**<.001**	.869	.820	.921
Fatherly warmth adolescence	-.120	.036	11.386	1	.001	.887	.827	.951
Fatherly control adolescence	-.027	.030	.832	1	.362	.973	.918	1.032
Age	.319	.023	186.224	1	**<.001**	1.376	1.315	1.441
Sex*	1.189	.039	950.163	1	**<.001**	3.283	3.044	3.540
Constant	-5.063	.385	173.007	1	**<**.001	.006		

*Coding: 1 = female.

In the second variant of the basic models where Baumrind’s four parenting style variables were used as predictors, the binary logistic regression (Chi^2^ (6) = 1849.358; p < .001) showed that three parenting styles were associated with suicide attempts. There was a positive association (in the sense of a higher probability of suicide attempts) with Authoritarian as well as with Rejecting-Neglecting parental behavior in childhood and later suicide attempts. By contrast, an Authoritative parenting style in childhood was associated with a lower probability of a lifetime history of suicide attempts (Table 2). In this model, again, the two control variables age and sex showed a significant correlation with the dependent variable, and the amount of explained variance was 9.1% (R^2^).

**Table 2:** Basic model: predictive values of four parenting style variables on suicide attempts (N = 44,134)

	**Regression coefficient β**	**Standard-error**	**Wald**	**df**	**p**	**OR**	**95% Confidence interval for OR**	
							**Lower value**	**Upper value**
Authoritative	-.559	.051	121.682	1	**<.001**	.572	.518	.631
Permissive	-.165	.122	1.829	1	.176	.848	.668	1.077
Authoritarian	.446	.088	25.712	1	**<.001**	1.562	1.315	1.856
Rejecting-Neglecting	.803	.040	401.710	1	**<.001**	2.233	2.064	2.415
Age	.360	.023	245.637	1	**<.001**	1.434	1.371	1.500
Sex*	1.100	.037	871.518	1	**<.001**	3.005	2.793	3.233
Constant	-8.610	.358	576.847	1	**<**.001	.000		

*Coding: 1 = female.

The comparison of the two basic models showed that there was not equivalence between the predictive values of parental behavior variables and parenting styles. Whereas the first basic model revealed only protective parenting variables, the second model also highlighted risky parental behavior next to protective factors as predictive of suicide attempts.

### Step II: bivariate models with basic control variables

A total of 13 variables were analyzed separately in single models (binary logistic regressions) for their ability to predict suicide attempts. Each model was again controlled for age and sex and also for the four parenting style variables. The goals were a) to identify potential predictors of suicide attempts in addition to parenting styles and b) to detect possible changes in the predictive power of the parenting style variables when including other potentially relevant predictors.

a) As a result, the models revealed that in addition to the four parenting styles, the following variables turned out to be statistically significant predictors of suicide attempts (each alone): migration background, welfare status, parental separation events, Binge Drinking, smoking, non-profit volunteer activities, school grades, social integration in school, absenteeism/truancy, attention deficit hyperactivity disorder, self-esteem, mental well-being/mood, school anxiety.

b) The following one variable changed the association of the parenting styles with suicide attempts: mental well-being/mood: p-Level of Authoritarian parenting style changed from p < .001 to p = .001.

### Step III - final: full model

In the last step (multivariate analysis), the four parenting styles and all significant predictors from Step II (see results of Step II) plus age and sex were included simultaneously in a multiple binary logistic regression (Table 3) with suicide attempts as the dichotomous dependent variable. The model (Chi^2^ (21) = 4530.968; p < .001) explained 21.7% (R^2^) of the variance.

**Table 3:** Full model: predictive values of parenting style variables and additional predictors on suicide attempts (N = 44,134)

	**Regression coefficient β**	**Standard-error**	**Wald**	**df**	**P**	**OR**	**95% Confidence interval for OR**	
							**Lower value**	**Upper value**
Authoritative	-.236	.053	19.779	1	**<.001**	.789	.711	.876
Permissive	-.088	.126	.495	1	.482	.915	.715	1.171
Authoritarian	.262	.093	7.855	1	.005	1.299	1.082	1.560
Rejecting-Neglecting	.488	.043	127.803	1	**<.001**	1.629	1.497	1.773
Age	.149	.025	34.646	1	**<.001**	1.160	1.104	1.219
Sex*	.980	.041	582.883	1	**<.001**	2.665	2.461	2.885
Migration background^§^								
I) Eastern Europe	.087	.058	2.294	1	.130	1.091	.975	1.221
II) Islamic imprinted countries	.437	.071	37.442	1	**<.001**	1.548	1.346	1.780
III) Other countries	.217	.055	15.538	1	**<.001**	1.243	1.116	1.385
Welfare status^$^	.107	.056	3.622	1	.057	1.113	.997	1.243
Binge Drinking last 4 weeks&	.566	.043	170.502	1	**<.001**	1.761	1.618	1.917
Smoking last 12 months&	.750	.042	325.125	1	**<.001**	2.118	1.952	2.298
Absenteeism/Truancy&	.447	.039	133.155	1	**<.001**	1.563	1.449	1.687
ADHD&	1.006	.052	369.800	1	**<.001**	2.735	2.469	3.031
Parental separation events&	.294	.038	59.122	1	**<.001**	1.342	1.245	1.447
Non-profit volunteer activities	.159	.021	57.330	1	**<.001**	1.172	1.125	1.221
School grades^£^	.172	.027	40.913	1	**<.001**	1.187	1.126	1.251
School anxiety	.041	.005	57.705	1	**<.001**	1.042	1.031	1.053
Mental well-being^~^	.104	.007	249.572	1	**<.001**	1.110	1.095	1.124
Self-esteem	-.051	.006	70.423	1	**<.001**	.950	.939	.961
Social integration in school	-.043	.012	12.752	1	**<.001**	.958	.935	.981
Constant	-7.652	.412	345.281	1	<.001	.000		

*Coding: 1 = female.

^§^in comparison to native German adolescents.

^$^Coding: 1 = living on welfare.

&Coding: yes = 1.

^£^School grades in Germany: 1 to 6; 1 = best performance; 6 = insufficient.

^~^Higher scores represent lower mental well-being.

Again, as found for the basic model, an Authoritative parenting style was significantly associated with suicide attempts in the sense of a protective effect, whereas (also again) the significant association of the Rejecting-Neglecting parenting style with suicide attempts constituted a risk factor. The Authoritarian parenting style was no longer a significant predictor of suicide attempts.

Other statistically significant (p < .001) and clinically relevant risk (OR ≥ 1.2) or protective (OR ≤ 0.8) factors were: Sex (with a higher association of suicide attempts for females), Migration background (higher association for adolescents from Islamic imprinted countries and other countries in comparison to “native” German adolescents), Binge Drinking, Smoking, Absenteeism/Truancy, ADHD, and Parental Separation events. Next to the parenting styles, all identified significant and relevant predictors were interpreted to be risk factors. Except for the Authoritative parenting style, there was no predictor that could be identified as a protective factor for suicide attempts.

Four additional predictors were on the threshold of clinical relevance and were still statistically significant (p < .001). In all of them, a higher value was associated with a higher probability of suicide attempts: age, number of non-profit volunteer activities, school grades (higher grade “numbers” constitute lower performance in the German school system), and impaired mental well-being.

**Figure 2:**
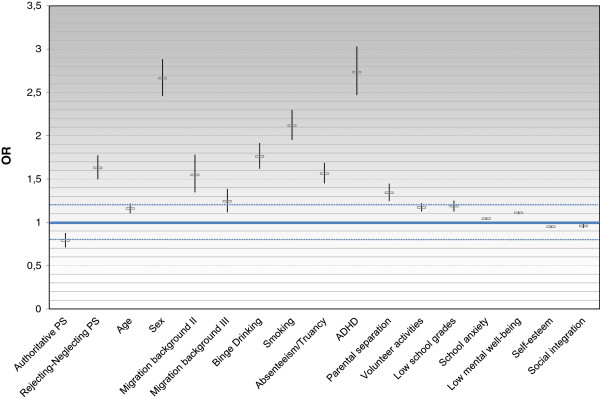
**Odds ratios including confidence intervals of statistically significant predictors (p < .001).** PS: Parenting style. Migration background II: Islamic imprinted countries. Migration background III: Other countries.

Differing from the results of the bivariate model, Welfare status was no longer significantly associated with suicide attempts in adolescents. An overview of all significant predictors of suicide attempts in adolescence is shown in Figure 2.

### Sensitivity analysis

As a sensitivity analysis, Step III was also computed with suicidal thoughts (dichotomous) as the dependent variable. Thus, the four parenting styles and all significant predictors from Step II plus age and sex were included simultaneously in a multiple binary logistic regression. The model (Chi^2^ (21) = 8814.640; p < .001) explained 24.6% (R^2^) of the variance. The results were basically the same as for the analysis with suicide attempts. Additionally, the Authoritarian parenting style was a significant predictor (p < .001; OR: 1.59), and on the other hand, school grades were not a significant predictor of suicidal thoughts (as opposed to suicide attempts): p = .083.

## Discussion

The aim of the study was to analyze the predictive value of parenting variables and parenting styles on suicide attempts in a representative sample of German adolescents. Furthermore, we aimed to identify statistically significant and clinically relevant protective and risk factors for suicide attempts in adolescents besides parenting styles. As a final result, in addition to the obviously relevant parenting styles, seven significant and clinically relevant risk factors for suicide attempts were identified.

Some of the findings of this work are in the expected direction and in accordance with the literature. Some of the findings are really new and have not been discussed in other studies so far. The following section discusses the state-of-the-art knowledge concerning the eight most relevant risk and protective factors for suicidal behavior in adolescents in comparison to the results of our study.

### Variables classified being significant and relevant

#### Parenting styles

The positive effect of Authoritative parenting was already proposed by Baumrind herself in 1966, but we still formulated this hypothesis [36]. As already shown for other risk behaviors [18], Authoritative parenting protects against suicidal behavior and was shown to lower the risk of suicide attempts by about 20% in our study. This was the only protective factor that could be identified in these analyses. Other studies have not explicitly classified parenting styles but have shown that parental social support and affection serve as factors that protect against suicide attempts [37-39]. Wichstrom showed in a predictor analyse that attachment to parents was protective against suicidal attempts [40]. A recent systematic review on interventions for suicidal prevention confirms the important protective role of positive family processes and suggests the augmentation of familial support for prevention [41].

Our results show a relatively new result concerning the role of Rejecting-Neglecting parenting and suicidal thoughts and attempts. Having Rejecting-Neglecting parents increases the risk of suicide attempts in adolescents by more than 1.5 times. Until now, only the risk factor of Authoritarian parenting has been discussed [19,20]. We confirmed this result for Authoritarian parenting in the basic model, but it was no longer significant in the full model. However, the association is definitely lower in comparison to the Rejecting-Neglecting parenting. We could confirm the role of Authoritarian parenting for suicidal thoughts in our sensitivity analysis.

#### Sex

Females are at higher risk of attempting suicide [23]. This well-researched fact [38,39,42-44] was also demonstrated in our study with German adolescents such that 15-year-old girls showed a 2½-fold higher probability of lifetime suicide attempts than boys.

#### Migration background

There is only sparse literature on the association of migration background and suicidal behavior. We found that being an adolescent with a migration background living in Germany was associated with a higher risk for suicide attempts especially for young people with roots in Islamic imprinted countries or other Non-European or Western/Southern European countries. There was one study from Asia that confirmed migration background as a risk factor at least for suicidal thoughts [42] and one study from the US that described a “cultural mix” as a risk factor for suicidality in adolescents [38]. As we know only a little about the health of adolescents with migration backgrounds (except for a higher risk for obesity and its consequences [45,46]), it seems necessary to continue researching this growing group and to adapt already existing prevention measures to the cultural backgrounds of adolescents. Obviously, the existing measures that are being implemented do not work with the same efficiency in immigrant groups as for “native” adolescents.

#### Binge drinking

In our study, engaging in Binge Drinking at least once in the last 4 weeks was associated with an almost doubled risk for lifetime suicide attempts (OR 1.76). The presence of depression and emotional problems are known to be positively associated with Binge Drinking [47]. Studies that have explicitly explored the association between suicidal thoughts/attempts and substance use have supported our results also, even though they were not all specifically aimed at Binge Drinking [48] but rather at alcohol use [49] or misuse in general [50-52].

#### Smoking

In our study, we found an association between legal tobacco use (i.e., smoking) and suicide attempts. It has to be kept in mind that 15-year-olds (i.e., the population of this study) are under the legal age for using tobacco in Germany. Smoking was a risk factor that more than doubled the risk for suicide attempts in this data set. The results of smoking as a risk factor for suicide attempts are supported by the literature [49,53,54].

#### Absenteeism/truancy

In our study, being regularly absent from school without an excuse (i.e., truancy/absenteeism) was a predictor of suicide attempts that raised the risk about 1½ times. This finding has not been discussed so far in the literature except for one Chinese study that found, in line with our study, that a higher number of days of unexcused absences was associated with suicidal thoughts/attempts [39]. The finding fits with the result of bad school grades as a predictor of suicide attempts [44] as in reality low school performance and absenteeism are often associated.

#### ADHD

We found a history of medically diagnosed ADHD to be a main risk factor for suicide attempts. After female sex, ADHD was the variable with the highest OR. Obviously, adolescents with ADHD seem to be a vulnerable group – first being diagnosed with conduct disorders (including externalizing behavior) in childhood and developing possible depressive symptoms later on – for which we have only the indicator of suicidal thoughts and attempts. There is a small-sample study from the US that supports our data – also describing ADHD as a risk factor for suicide attempts [55]. It is possible that the impulsivity that is a part of ADHD allows suicidal thoughts to more quickly advance to a suicide attempt. There are hints that impulsivity is a predictor of suicidal thoughts [56] next to externalizing behavior [57]. It seems worthwhile for prevention measures to focus on this rather small but highly relevant group in health services research. It is possible that this subgroup will develop even more mental problems or will continue to harm themselves if no intervention is implemented.

#### Parental separation

The risk of adolescent suicide attempts due to parental separation has not been discussed very much so far. Bolognini and colleagues propose “loss” as a risk factor for suicide attempts [52], and a study from Turkey showed that having divorced or widowed parents constitutes a risk factor mainly for male adolescents [44]. As our study suggests suicide attempts in adolescents can be associated with experiences of parental divorce or loss and therefore with changes in one’s family and one’s social support system, which can be seen as critical life events. This result is supported by one Chinese study [58] and one US study that found significant associations between displacement, belonging, and suicidal behavior [59].

### Variables classified being below clinical relevance (or being not significant)

#### Welfare status

The economic situation of the family in our study was one of the rare non-significant predictors. For adolescents in Germany, having a family living on welfare was not a risk factor for suicide attempts. This stands in contrast to the one study from India that reported results on this variable [43].

#### Self-esteem

In the literature, low self-esteem is discussed as a risk factor for suicide attempts [37,44]. We found a significant protective effect against suicide attempts for high self-esteem; however, it remains below the threshold of clinical relevance.

#### School grades

As in the study by Eskin and colleagues [44], in our study, school grades were significantly associated with suicide attempts such that worse grades predicted a higher risk for suicide attempts. These results are supported by a Korean study in which low academic achievement was a risk factor for suicide attempts [49].

#### Mental well-being

We found that low mental well-being was associated with suicide attempts as one would expect. Other researchers have not reported on mental well-being but on depressive symptoms [37,44] or on a low “happiness level” [49] as positively associated with the risk for suicide attempts.

#### Age

In our study, age was a statistically significant risk factor but fell short of the defined level of clinical relevance. As other studies have already shown, older age of the adolescents was associated with higher risk for the lifetime prevalence of suicide attempts [38,39,42].

#### Non-profit volunteer activities

Against the expectations, volunteering in the spare-time was not a significant protective factor against suicidal attempts rather it seemed here to be adversely associated. Since we do not have longitudinal data we cannot explain this. However it could be that the adolescents having a suicidal attempt in the past are now recovering and engage in activities. Still interpretation remains unclear.

### Limitations

All data analyzed in this study rely on self-stated information. This could be a source of bias. In future studies a verification of central constructs should be aimed. As always with self-stated data referring to personally sensitive information persons tend to give social acceptable answers. This could have led to an underestimation of social questionable behavior like substance consumption or suicidal attempts. Concerning the assessment of suicidality the measurement (single items) was new and information about the number of suicidal attempts was not included. Furthermore, as depression is a well-known risk-factor for suicidality, a limitation of the study is that life-time depression diagnoses could not be included into the analyses because of the weak data quality of this item.

## Conclusions

Parenting style does matter. Whereas children of Authoritative parents profit, children of Rejecting-Neglecting parents are put at risk – as we found for suicide attempts in adolescence. As these findings apply not only to suicidal behavior but also to other risk behaviors, early and wide-spread information to new parents on parenting might be a helpful option. Such information should teach parents about and advocate for Authoritative parenting. Rather difficult but still possible would be to try to change parenting styles by implementing intervention programs aimed at parents who had developed Rejecting-Neglecting parenting as a coping-strategy over time because “difficult” children had placed an excessive demand on them. From the perspective of the adolescent, some of identified risk factors for suicide attempts are not surprising (e.g., sex), whereas others contribute new knowledge and indicate possible points of intervention for child or adolescent prevention programs. This suggests adaptions of already existing interventions (e. g. for substance consumption) to include contents on suicidal behavior; and furthermore adaptions of suicide prevention programs for special groups such as migrants or children diagnosed with ADHD. Further studies should evaluate the effect of intervention programs for these groups on suicidal attempts.

## Competing interests

The authors declare that they have no competing interests.

## Authors’ contributions

CD carried out the data analyses and drafted the manuscript. DB worked out parts of the Methods section. EG suggested the three-step analytical model and contributed to the Discussion section and to the conclusions. TH and SB organized the study and revised the manuscript. All authors read and approved the final manuscript.

## Pre-publication history

The pre-publication history for this paper can be accessed here:

http://www.biomedcentral.com/1471-2431/14/113/prepub
